# Family caregivers’ perspectives on caring for mentally ill adult patients in tertiary hospital in Khyber Pakhtunkhwa, Pakistan

**DOI:** 10.12669/pjms.41.8.12003

**Published:** 2025-08

**Authors:** Somia Saghir, Anny Ashiq Ali, Jalal Khan, Farhana

**Affiliations:** 1 Somia Saghir, PhD Student, MSN Assistant Professor, Shifa College of Nursing, Shifa Tameer-e-Millat University, Islamabad, Pakistan; 2 Anny Ashiq Ali, MSN Assistant Professor, IQRA University Nursing College, Karachi Pakistan; 3 Jalal khan, MSN Assistant Professor, Bilal institute of Nursing and Health Sciences, KPK, Pakistan; 4 Farhana, MSN Senior Lecturer, Shifa College of Nursing, Shifa Tameer-e-Millat University, Islamabad, Pakistan

**Keywords:** Experiences, Family caregivers, Informal caregivers, Mental health caregiving, Perspectives

## Abstract

**Background & Objective:**

Mental health disorders affect approximately 450 million individuals globally, with a significant burden observed in Pakistan. The country faces a critical shortage of mental health professionals, leaving family caregivers (FCGs) as the primary source of care for individuals with psychiatric illnesses. This study aims to explore the experiences of family caregivers providing care for mentally ill adult patients admitted to tertiary care hospital in Khyber Pakhtunkhwa (KPK), Pakistan.

**Methodology:**

A qualitative exploratory descriptive study was conducted from October 2024 to January 2025 with family caregivers of mentally ill patients in KPK, Pakistan. Convenience sampling was used to recruit 18 participants from a private tertiary care hospital. In-depth interviews were conducted using semi structured interview guide. Content analysis was conducted using Creswell and Creswell’s five-step inductive approach to identify emerging themes and categories.

**Results:**

Findings revealed that caregivers experience a “silent struggle” characterized by internal and external factors. Internal factors include emotional and psychological burdens such as stress, anxiety, and burnout. External factors encompass financial strain, inadequate training and knowledge, healthcare system barriers, and social stigma. Caregivers reported feeling unprepared for their roles, struggling with financial instability due to reduced employment opportunities, and facing difficulties navigating the healthcare system. Additionally, societal stigma further isolates caregivers, limiting their access to social support and exacerbating their emotional distress.

**Conclusion:**

The study emphasizes the need for structured support for caregivers through training, financial aid, and better healthcare access. Policy reforms and community initiatives can enhance caregiver well-being and care quality. Future research should explore interventions to reduce caregiver burden, especially in resource-limited settings.

## INTRODUCTION

Mental health disorders affect around 450 million people globally, with nearly half of these cases beginning before the age of 14,^1^ unfortunately, more than 160 million people require mental health support following crises.^2^ These figures emphasize the increasing impact of mental illnesses and highlight the vital role caregivers play in assisting patients.

In Pakistan, nearly 24 million people require psychiatric care, as mental health disorders significantly add to the overall disease burden.^3^ However, the country faces a severe shortage of mental health professionals.^4^ In many regions, the disparity is even greater, with only 2.19 psychiatrists and 5.51 nurses available for every 100,000 people.^5^ Family caregivers (FCGs) play a crucial role in supporting individuals with mental illnesses, particularly in Pakistan, where mental health resources are scarce.^6^ Caregivers of individuals with mental illnesses provide multifaceted support, encompassing assistance with daily living activities such as bathing, dressing, grooming, feeding, and toileting-as well as managing essential responsibilities like financial matters.

They also perform nursing duties, including administering medications and facilitating treatments like electroconvulsive therapy (ECT) and post-ECT care.^7^ This shortage of healthcare professionals exacerbates the burden on FCGs, who often assume responsibilities that would typically fall to mental health professionals.^8^ Psychiatric disorders contributes to over 6% of the country’s total disease burden,^9^ and the lack of sufficient healthcare infrastructure and professional support leaves many FCGs without the necessary guidance or training. This leads to heightened emotional and physical stress for caregivers.^10^

Moreover, cultural stigma and a lack of mental health awareness often create barriers to care, causing many individuals to avoid or postpone seeking professional help—ultimately worsening their condition and delaying effective treatment.^11^ Research indicates that while approximately 50 million Pakistanis will experience mental health issues during their lifetime,^4^ nearly 90% of those requiring treatment do not receive it due to stigma and poor healthcare access.^12^ Consequently, caregivers face increased stress, isolation, and frustration, as they often manage caregiving responsibilities without adequate external support.^11^ This lack of structured support mechanisms forces caregivers to navigate these challenges on their own, further contributing to high levels of stress, anxiety, depression, and chronic fatigue.^13^

The caregiving role can also disrupt work-life balance and social interactions, leading to burnout and diminished quality of life for caregivers.^14^ Despite the growing recognition of the importance of mental health, Pakistan’s limited resources and infrastructure hinder the country’s ability to meet the increasing needs of individuals with psychiatric disorders.^15^ In light of these challenges, many FCGs not only provide care at home but also assist within hospitals due to nursing shortages and extended patient stays.^16^ To better support caregivers, it is critical to explore their experiences in depth. Understanding their challenges, coping strategies and support needs is vital for improving healthcare systems and caregiver well-being.^17^ Therefore, the objective of this study was to explore the experiences of family caregivers responsible for providing care to mentally ill adult patients admitted to tertiary care hospital of KPK, Pakistan.

### Research Question:

What are the experiences of family caregivers providing care for mentally ill adult patients admitted to tertiary hospital of KPK, Pakistan?

## METHODOLOGY

A qualitative exploratory descriptive study was conducted from October 2024 to January 2025 at Medrect International Hospital Timergara from October-December.

### Ethical Approval:

It was obtained from Institutional Review Board (IRB) (Ref. No. ERC 18300, Dated: October 1, 2024).

### Participants and setting:

Participants were selected through convenience sampling, focusing on caregivers aged 18 years and above who were responsible for family members with mental illnesses and proficient in English or Urdu. The study sample was collected from a private sector tertiary care hospital in KPK. Individuals caring for persons with non-psychiatric conditions were excluded.

### Data collection:

Following IRB approval and institutional permission, the researcher obtained informed consent after explaining the study’s purpose, risks, and benefits. Semi-structured in-depth interviews with open-ended questions explored family caregivers’ experiences. A mock interview refined the guide before formal interviews. Face-to-face interviews (25-45 minutes) were audio-recorded, transcribed verbatim, and supplemented with field notes on non-verbal behaviors. Confidentiality was ensured by assigning unique codes.

### Population:

The study included 18 caregivers of adult individuals living with mental illness. The interview began with demographic information, including the caregiver’s age, gender, the diagnosis of their family member, their relationship to the ill family member, educational status, and employment situation.

### Data analysis:

Qualitative interviews were transcribed, manually coded, and verified for accuracy. Data analysis followed^18^ five-step inductive approach: organizing data, repeatedly reviewing transcripts, coding key content, grouping similar codes into categories, and developing descriptive narratives with participant quotes. Both manifest and latent content were analyzed collaboratively.

### Trustworthiness:

To ensure trustworthiness,^19^ criteria was followed. Audio recordings were compared with transcriptions, while two independent experts verified coding. Detailed descriptions of the research context and participants is given.

## RESULTS

The sample included 18 caregivers, with a majority being female (83%) and primarily housewives (67%), with ages spanning from 27 to 58 years. The caregiving period ranged from 3 to 20 years, with the most common family mental disorders being schizophrenia (33%) and bipolar disorder (17%). Caregivers’ educational levels varied, with 50% having completed secondary or higher education. Most caregivers reported providing care to spouses or children, with significant variations in caregiving duration and mental health conditions. The specific psychiatric diagnoses of the care recipients are detailed in Table-I.

**Table-I:** Demographic Distribution.

Caregiver Identifier	Age	Gender	Marital status	Occupational status	Educational level	Relationship with Participant	Family Member’s Mental Disorder	Caregiving period
FCG 1	45	Female	Married	Housewife	Primary	Husband	Bipolar, depression	10 years
FCG 2	55	Female	Married	Housewife	Primary	Son	Obsessive-compulsive disorder, depression	5 years
FCG 3	54	Female	Married	Housewife	Primary	Husband	obsessive-compulsive	8 years
FCG 4	53	Female	Married	Housewife	Primary	Daughter	Depression	3 years
FCG 5	27	Female	Single	Student	High school	Sister	Suicidal ideation	9 years
FCG 6	45	Male	Single	Employed	Bachelors	Brother	Depression, Bipolar	10 years
FCG 7	47	Female	Married	Employed	Bachelors	Wife	Depression	4 years
FCG 8	25	Female	Single	Student	High school	Mother	Alzheimer’s	10 years
FCG 9	45	Female	Married	Employed	High school	Sister in Law	Bipolar	9 years
FCG 10	54	Male	Married	Employed	Bachelors	Mother	Alzheimer’s	9 years
FCG 11	54	Female	Married	Housewife	High school	Son	Autism	20 years
FCG 12	43	Female	Married	Employed	High school	Son	Schizophrenia	7-8 years
FCG 13	49	Female	Married	Housewife	High school	Mother in Law	Schizophrenia	5-7 years
FCG 14	58	Male	Married	Employed	Masters	Daughter	Autism	15 years
FCG 15	55	Female	Married	Employed	Bachelors	Husband	Intellectual Disability	10 years
FCG 16	48	Female	Married	Housewife	Masters	Mother in Law	Schizophrenia	5 years
FCG 17	55	Male	Married	Employed	Masters	Brother	Schizophrenia	10-11 years
FCG 18	57	Female	Married	Housewife	Bachelors	Father in Law	Schizophrenia	5 years

**Fig.1:**
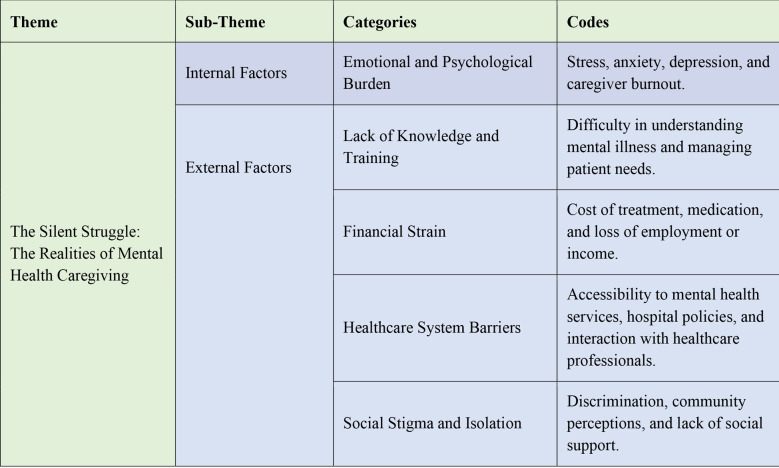
Theme, Sub-themes, Categories and Codes.

The findings are organized into a main theme, two sub-themes, and five categories (Fig.1), each supported by participant quotes. Minor grammatical adjustments were made for clarity while preserving intent. Anonymity was ensured using participant codes (e.g., FCG1, FCG2).

### Theme, Sub-theme, Categories and Codes:

### The Silent Struggle – The Realities of Mental Health Caregiving:

In the analysis of the caregiving experience for mentally ill adults, two primary themes emerged: Internal Factors and External Factors. These interconnected challenges create an invisible struggle, emphasizing the need for awareness, support, and stigma reduction.

### Subthemes: Internal Factors

### Emotional and Psychological Burden:

Caring for a mentally ill adult can take a significant emotional toll on caregivers, leading to stress, anxiety, and burnout. Many caregivers experience a persistent state of worry and emotional exhaustion. *“There are days when I feel completely drained, emotionally and physically. I love my brother, but his condition leaves me feeling helpless and overwhelmed”* (FCG 4). “*It feels like I’m carrying the weight of the world, overwhelmed by constant stress with no time for myself*” (FCG 15).*”I barely sleep at night, constantly fearing that something might happen. The stress is unbearable, but I don’t have the option to step back”* (FGC 9). “*I feel lost in this journey, consumed by caregiving, wondering if anyone notices the toll it takes on me*” (FCG 12).

### Subthemes: 2. External Factors

### Lack of Knowledge and Training:

Caregivers often struggle due to a lack of formal training on how to manage the needs of mentally ill patients. The absence of proper guidance leads to uncertainty in handling crises. *“No one teaches you how to care for a mentally ill person. I had to learn everything through trial and error, which is frustrating and scary”* (FCG 5). *“The hospital staff gives instructions, but they assume we know what to do. I often feel unprepared when my sister has an episode”* (FCG 8). *“* *I often feel lost handling my brother’s behavior and wish there were more resources or guidance for these situations”* (FCG 18).

### Financial Strain:

The financial burden of caregiving is a major challenge, particularly for those who have had to reduce their working hours or leave their jobs entirely, leaving them overwhelmed. *“I quit my job to care for my mother, leaving us in financial hardship with no aid for costly treatment”* (FCG 2). **“**We sacrifice our own needs to afford my son’s costly medication each month” (FCG 7). *“Even with part-time job I am unable to cover the cost of my wife’s treatment and medication. It feels like we’re drowning financially”* (FCG 11).

### Healthcare System Barriers:

FCGs struggle with accessing quality mental health services and navigating bureaucratic hurdles. *“The hospital staff is overburdened, and sometimes they do not have time to answer our questions. We are left figuring things out on our own”* (FCG 3). *“Getting an appointment with a psychiatrist takes months. By the time we get help, the situation at home has already worsened”* (FCG 10). *“During my father’s ECT sessions, I was given no clear instructions. I had to beg the staff for information, and even then, no one properly guided me on what to expect”* (FCG 13).

### Social Stigma and Isolation:

Mental illness is highly stigmatized, causing FCGs to face social isolation and discrimination, often feeling alienated due to misconceptions. *“People treat us differently after my son’s mental sickness. Some even avoid us, as if mental illness is contagious”* (FCG 1). *“I used to have a strong support network, but now most of my friends have distanced themselves. They don’t understand what I go through”* (FCG 6). “*Even my relatives tell me to keep my husband’s illness a secret. They say it will ruin our family’s reputation”* (FCG 11). *“I try to avoid talking about my sister’s condition because I know people will judge me. It feels like I am constantly hiding a part of my life”* (FCG 14).

## DISCUSSION

The findings of the study include caregivers’ silent struggle underpinned by internal and external factors. It also highlights the multifaceted challenges faced by family caregivers of mentally ill adults and the recommendations for supporting these caregivers are identified as the critical issue requiring immediate intervention. The result revealed the internal factor surrounding the emotional burden on caregivers as many of the participants reported challenges related to stress, anxiety, and burnout. These findings align with recent studies that indicate caregivers of mentally ill patients face emotional distress.^20^ Moreover, studies also focus on Asian caregivers who manifest more prominent symptoms of emotional stress as compared to Western settings. However, this tends to exacerbate the burden on caregivers and impact their psychological well-being and quality of life.^13^ Another study also coincides with the findings, that caregivers of schizophrenia patients had high levels of psychological distress due to uncertainty of their patients’ acts.^21^ Similarly, in this study caregivers also expressed their feelings of helplessness, exhaustion, and developing a mental health support system unique to the needs of caregivers.

The lack of knowledge and training among caregivers was another important finding of the study. Participants often felt unprepared and unaware of the situation and managed the crisis with insufficient knowledge and training. This finding is consistent with content shared in the book, where family caregivers reported that inadequate training becomes the barrier to effective care provided to mentally ill patients.^22^ Studies also compared that in Pakistan settings, caregivers experience additional challenges due to cultural factors including societal stigma and lack of formalized caregiver training programs as compared to Western settings. Western studies often report the availability of institutional support and financial assistance.^23^

As in our study participants learn through trial-and-error methods, which makes them frustrated and helpless. Along with that, one Pakistani study also emphasized that caregivers experience financial hardship and social isolation, and the strain often exacerbates psychological distress. These findings illustrate the need for focused support and interventions to reduce caregiver burden in the care of people.^14^ Therefore, Pakistan need urgent educational initiatives aimed at caregiver competency. Financial stress was another factor and main issue among caregivers in this study. Most caregiver has to sacrifice their work or quit their jobs to care for their family members. This finding is resonant with a study that highlighted the economic burden faced by families caring for individuals with mental illness.^24^ Furthermore, challenges in the health care system like service accessibility and bureaucratic inefficiencies. One of the studies also reflects the challenges faced by caregivers in navigating the healthcare complex.^16^ These systematic issues highlight the importance of policy reforms to improve and support mental health services.

One of the important external factors is the stigma associated with mental illness which is faced by the patients and it also brings isolation for the caregivers. Participants report social isolation and being discriminated against due to misconceptions about mental illness. This aligns with the study which reflected that these stigmas prevent more people from taking the step and seeking help and create discrimination and judgment of people, leading to an exaggeration of particular mental disorders.^8^ By addressing an under-researched area, this study provides context-specific insights into the emotional, financial, and systemic challenges faced by family caregivers of mentally ill adults in Pakistan. It adds to the global literature by emphasizing the clinical importance of integrating caregiver support into mental health services to enhance outcomes for both caregivers and patients. Moreover, the study captures powerful first-hand narratives, offering deep insight into the struggles of caregivers in a low-resource, culturally unique setting.

### Limitations and Recommendation:

Data were collected solely from a private healthcare setting, limiting insights into the experiences of caregivers in public sector facilities. To address these gaps, culturally tailored training, psychological support, and financial aid should be extended across both private and public sectors. Community awareness efforts are also needed to reduce stigma and build supportive networks for caregivers.

## CONCLUSION

This study highlights the emotional, financial, and systemic challenges faced by family caregivers of mentally ill adults in Pakistan. Tailored interventions could significantly improve care quality for both patients and caregivers. Global comparisons show that caregiver struggles differ due to cultural and systemic factors. Future research should focus on developing culturally appropriate support programs. These programs must address the unique caregiving issues in Pakistan.
